# Supplementary material for: Optimisation of an nIR-Emitting Benzoporphyrin Pressure-Sensitive Paint Formulation
Source: Sensors (Basel). 2025 Jul 23;25(15):4560. doi: 10.3390/s25154560 (PMC12349349; doi:10.3390/s25154560)
Supplement: Supplementary file 1 [file sensors-25-04560-s001.zip › sensors-3745996-supplementary.pdf]

Supporting Information

Optimisation of a NIR-emitting Benzoporphyrin Pressure-Sensitive Paint Formulation

Table of Contents

1. PSP *A-Priori* calibrations 1

2. Example plots of luminescence intensity response to temperature at 100 kPa 4

3. References 4

1. PSP *A-Priori* calibrations

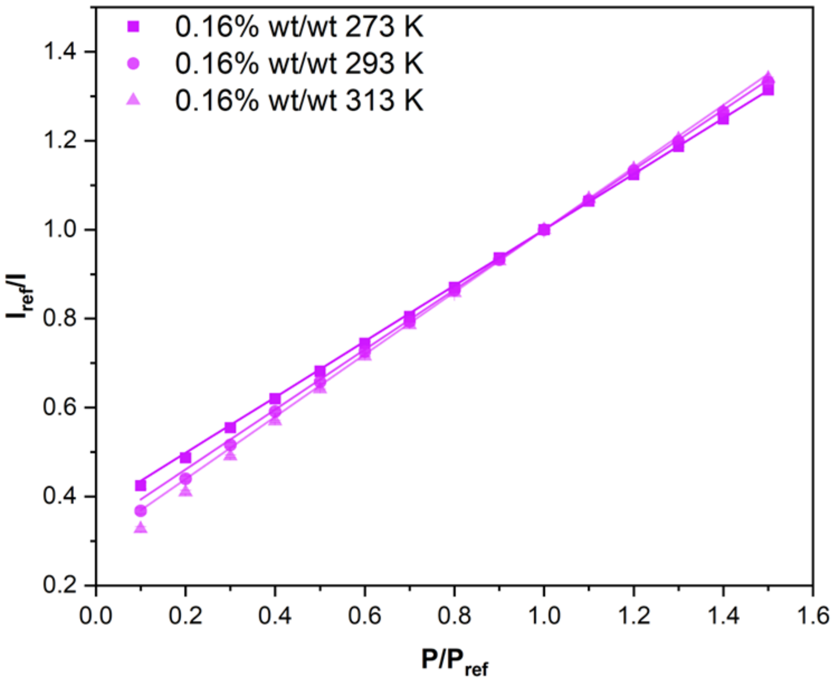

Fig. S1 The modified Stern-Volmer calibrated luminescence response with associated linear fits for the benzoporphyrin loading of 0.16% wt/wt at 273, 293 and 313 K.

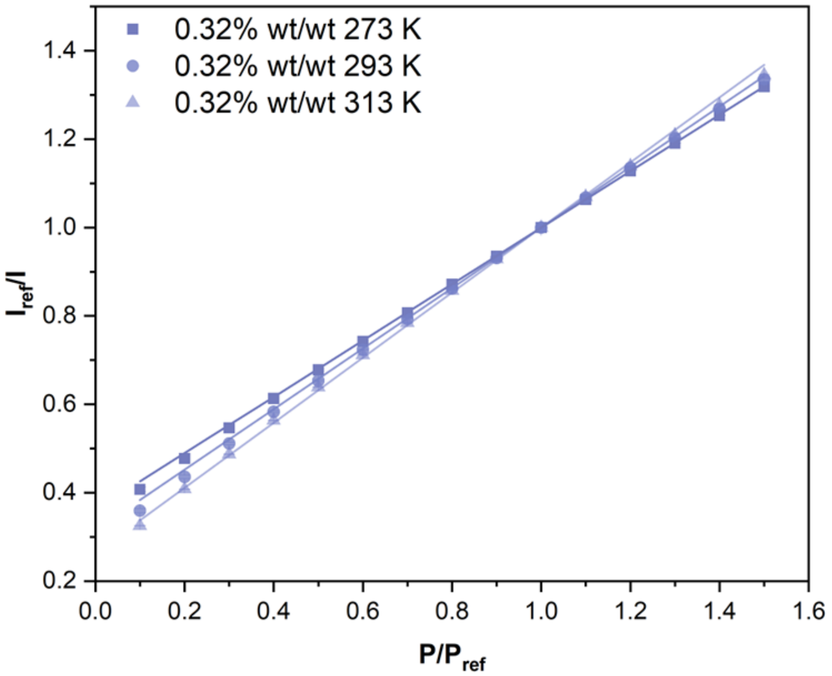

Fig. S2 The modified Stern-Volmer calibrated luminescence response with associated linear fits for the benzoporphyrin loading of 0.32% wt/wt at 273, 293 and 313 K.

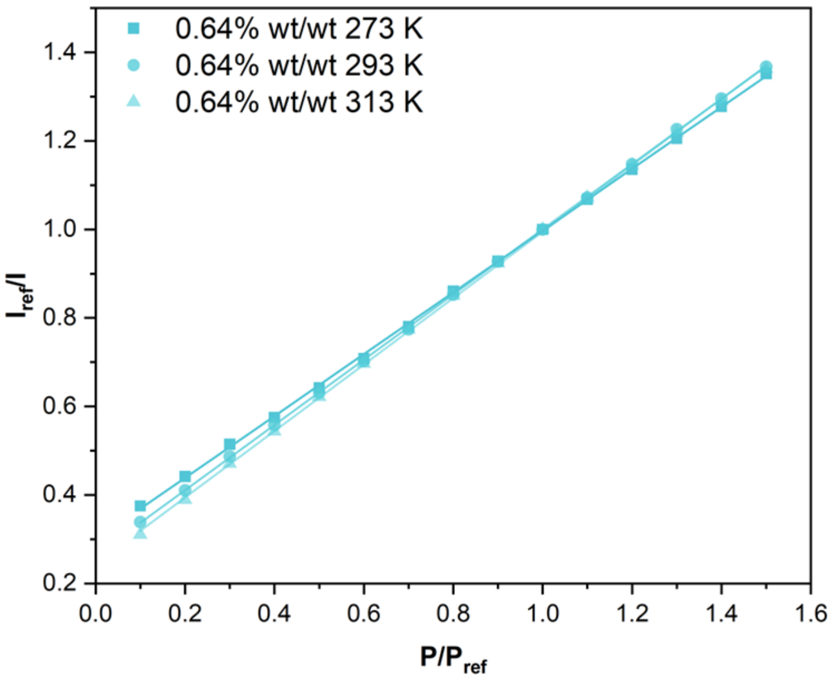

Fig. S3 The modified Stern-Volmer calibrated luminescence response with associated linear fits for the benzoporphyrin loading of 0.64% wt/wt at 273, 293 and 313 K. This data was published previously.[1]

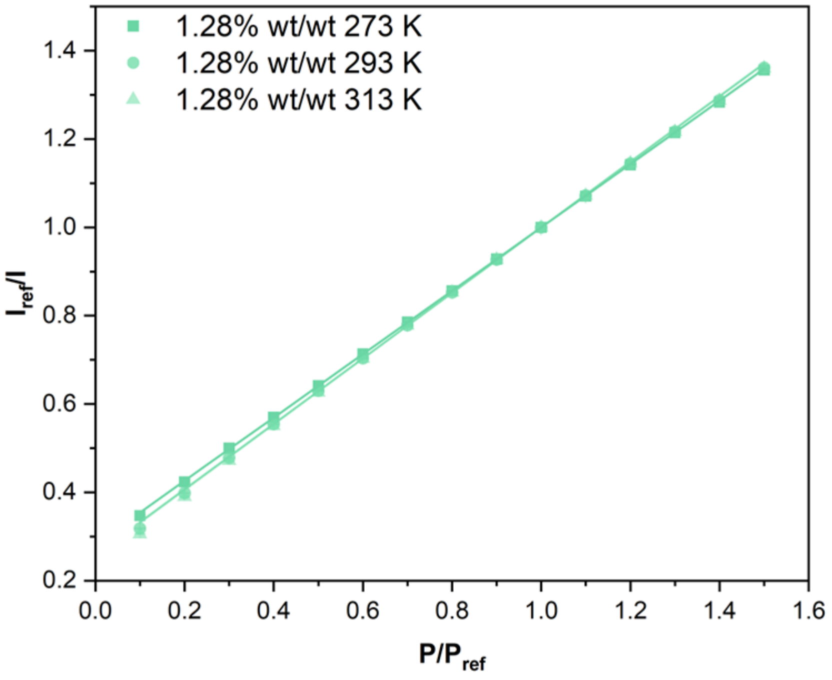

Fig. S4 The modified Stern-Volmer calibrated luminescence response with associated linear fits for the benzoporphyrin loading of 1.28% wt/wt at 273, 293 and 313 K.

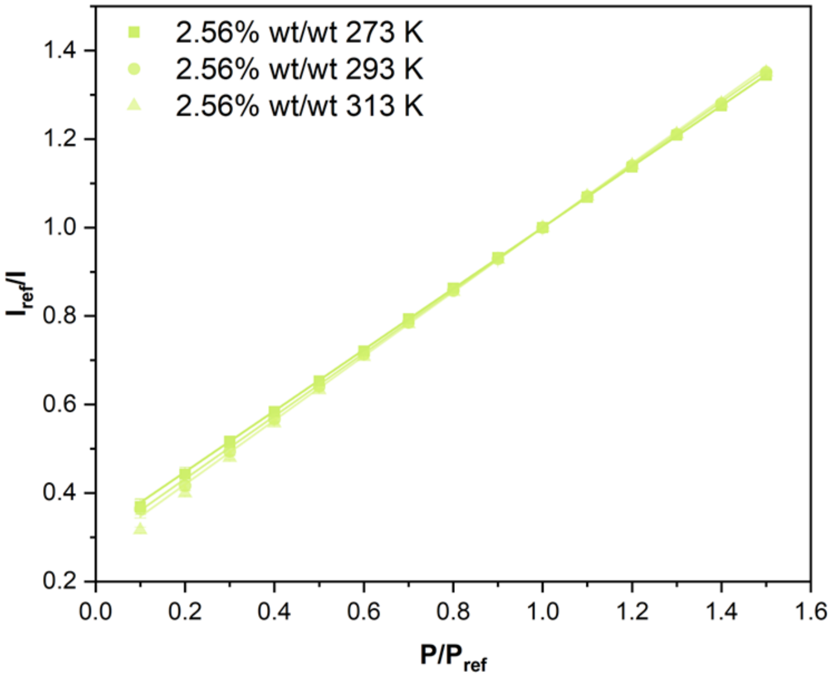

Fig. S5 The modified Stern-Volmer calibrated luminescence response with associated linear fits for the benzoporphyrin loading of 2.56% wt/wt at 273, 293 and 313 K.

2. Example plots of luminescence intensity response to temperature at 100 kPa

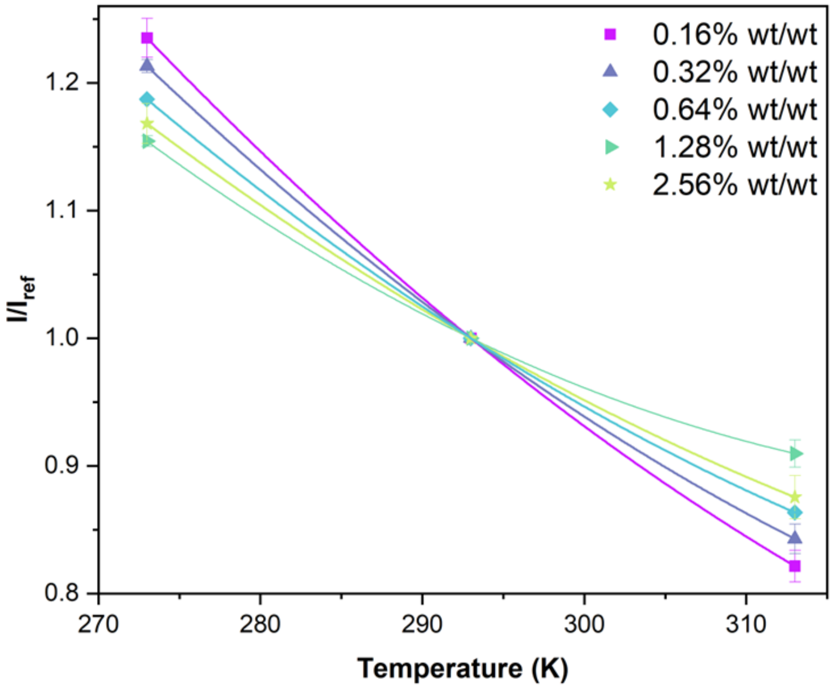

Fig S6. The plot of average I/I_{ref} with increasing temperature for the different benzoporphyrin loading BP-PSPs across three repeated samples with associated standard errors. I_{ref} is the luminescent intensity at 293 K and 100 kPa. S_T(100 kPa) is calculated as the slope of the plot at 293 K.

3. References

1. E. J. Nunn, D. Tsioumanis, T. B. Fisher, D. A. Roberts, M. K. Quinn and L. S. Natrajan, *Chem. Sci.*, 2025, **16**, 7018–7025
